# LINC01094 triggers radio-resistance in clear cell renal cell carcinoma via miR-577/CHEK2/FOXM1 axis

**DOI:** 10.1186/s12935-020-01306-8

**Published:** 2020-06-24

**Authors:** Yufeng Jiang, Wei Li, Yang Yan, Xudong Yao, Wenyu Gu, Haimin Zhang

**Affiliations:** 1 Department of Urology, Chongming Branch, Shanghai Tenth People’s Hospital, Tongji University School of Medicine, No.66 Xiangyang Road, Chongming District, Shanghai, 202157 China; 2 Department of Urology, Shanghai Tenth People’s Hospital, Tongji University School of Medicine, No.301 Yanchang Road, Jing’an District, Shanghai, 200072 China

**Keywords:** LINC01094, ccRCC radio-resistance, miR-577, CHEK2, FOXM1

## Abstract

**Background:**

Radioresistance is an obstacle to limit efficacy of radiotherapy. Meanwhile, long non-coding RNAs (lncRNAs) have been reported to affect radioresistance. Here, we aimed to investigate lncRNAs involving radioresistance development of clear cell renal cell carcinoma (ccRCC), the most frequent type of renal cell carcinoma (RCC).

**Methods:**

The mRNA and protein expressions of genes were measured via qRT-PCR and western blot. The relationships among genes were verified by RIP and luciferase reporter assay. The radioresistance of ccRCC cells was evaluated through clonogenic survival assay, MTT assay and TUNEL assay.

**Results:**

LINC01094 was over-expressed in ccRCC cell lines. LINC01094 expression was increased along with the radiation exposure time and the final stable level was 8 times of the initial level. Knockdown of LINC01094 resulted in enhanced radiosensitivity of ccRCC cells. Mechanically, LINC01094 was a ceRNA of CHEK2 by sponging miR-577. Also, the enhancement of LINC01094 on ccRCC radioresistance was mediated by CHEK2-stabilized FOXM1 protein.

**Conclusion:**

LINC01094 facilitates ccRCC radioresistance by targeting miR-577/CHEK2/FOXM1 axis, blazing a new trail for overcoming radioresistance in ccRCC.

## Background

Renal cell carcinoma (RCC) is one of the most aggressive cancers and accounts for 3% of all adult malignancies [1]. Clear cell renal cell carcinoma (ccRCC), characterized by a high rate of metastasis and relapse [2], is the most common subtype of RCC and represents approximately 80–90% of all RCC cases [3, 4]. Importantly, patients with metastatic ccRCC make up over 30% of all ccRCC cases and the 5-year survival rate of them was lower than 20% due to the resistance to chemotherapy and radiotherapy, [5, 6]. Although molecular characterization of ccRCC has got developed [7], the mechanism by which ccRCC patients obtain radioresistance or chemoresistance remains largely uncharted.

Radiotherapy is a commonly-applied cancer treatment as ionizing radiation (IR) damages cancer cell mainly via inducing DNA damage, especially DNA double strand breaks (DSBs) [8, 9]. The response of cancer cells to DNA damage is critical for tumor development, and enhanced repair on DNA DSBs results in resistance to IR [10]. In the past few decades, understanding of cellular signaling for DSBs repair has been gradually uncovered [11, 12]. Also, implication of non-coding RNAs (ncRNAs) in this process has been the focus on cancer research [13, 14].

Long non-coding RNAs (lncRNAs) are ncRNA transcripts with a length longer than 200 nts [15]. Recent studies indicated that lncRNAs play pivotal roles in the development of cancer radioresistance [16]. For example, SNHG18 improves radioresistance in glioma via inhibiting semaphorin 5A [17]. PCAT-1 regulates DSBs repair through repressing BRCA2 in prostate cancer [18]. Long intergenic non-protein coding RNA 1094 (LINC01094) is a lncRNA that has been scarcely explored before. Here, the TCGA data revealed that it was significantly upregulated in KIRC (Kidney renal clear cell carcinoma) tissues relative to the normal tissues. Therefore, we wondered whether LINC01094 was implicated in radioresistance development of ccRCC.

In the current study, we probed into the role and potential mechanism of LINC01094 in ccRCC radioresistance.

## Materials and methods

### Cell culture

Human kidney proximal tubule cell (HK-2), human embryonic kidney cell (HEK-293T) and ccRCC cells (A-498, ACHN, 786-O, Caki-1) were purchased from American Type Culture Collection (ATCC; Manassas, VA, USA). All cells were cultivated in RPMI-1640 medium (Invitrogen, Carlsbad, CA, USA) adding 10% fetal bovine serum (FBS; Invitrogen) plus 1% penicillin/streptomycin (Thermo Fisher Scientific, Grand Island, NY, USA) in a 5% CO_2_ atmosphere at 37 °C.

### Quantitative real-time PCR (qRT-PCR)

RNA was extracted using TRIzol reagent (Thermo Fisher Scientific) and then reversely transcribed into cDNA. SYBR Green PCR Master Mix (Roche, Mannheim, Germany) was applied on an Applied Biosystems 7900 Real-Time PCR System (Thermo Fisher Scientific) for real-time PCR. Relative RNA expression levels were assessed via 2^−ΔΔCt^ method. GAPAH/U6 acted as normalized gene.

### Cell transfection

786-O or Caki-1 cells with irradiation treatment or not, were firstly added into six-well plates with non-serum culture medium for 1 day. The specific shRNAs for LINC01094 (shLINC01094#1/2) or non-specific control (shCtrl), miR-577 mimics, miR-NC were synthesized by RiboBio (Guangzhou, Guangdong, China). Besides, the pcDNA3.1 vector targeting LINC01094, CHEK2 or FOXM1 and empty vectors were constructed by Genechem (Shanghai, China). Lipofectamine 2000 (Invitrogen) was used during transfection. Cells were collected after 48 h of transfection.

### Colony formation assay

Cells were plated into 6-well culture plates with a concentration of 800 cells per well. 14 days later, colonies were fixed for 15 min in 100% methanol (Sigma-Aldrich, St. Louis, MO, USA) and then stained for 20 min using 0.1% crystal violet (Sigma-Aldrich) at room temperature.

### MTT assay

Transfected 786-O or Caki-1 cells were seeded into 96-well plates with 4 Gy of irradiation treatment, culturing for 0, 24, 48, 72 and 96 h. Proliferation of cells was tested via MTT assay. 10 µL of MTT reagent (Sigma-Aldrich) was added to each well for 4 h followed by DMSO incubation. Absorbance at 490 nm of cell lysates was detected via a plate reader (Bio-Tek Instruments, Winooski, VT, USA).

### Immunofluorescence (IF) analysis

Cells were cultured on glass coverslips and fixed in in formaldehyde (Sigma-Aldrich), followed by permeabilization in cold methanol/acetone reaction. Afterwards, cell lines were treated with FITC-labeled phalloidin (Sigma-Aldrich) or primary antibody against phospho-Histone H2AX (ab2893; Abcam, Cambridge, USA), following treatment with Alexa Fluor488-labeled or Alexa Fluor594-labeled (Thermo Fisher Scientific) secondary antibody at room temperature for 1 h. Nuclei staining was conducted with 4′,6-diamidino-2-phenylindole (DAPI; Thermo Fisher Scientific). Nikon Eclipse E600 microscope and ACT-1 software (Nikon) were finally utilized.

### Nuclear-cytoplasmic extraction assay

Nuclear and cytoplasmic proteins were extracted from Caki-1 or 786-O cells using the NE-PER kit (Thermo Fisher Scientific). The qRT-PCR was used for the detection of LINC01094. GAPDH was used as an indicated reagent to detect the gene expression level in cytoplasm, while U6 was used to detect the expression level in nucleus.

### RNA immunoprecipitation (RIP)

The Imprint RNA Immunoprecipitation Kit (Millipore, Bedford, OH, USA) was applied for RIP assay. Briefly, cell extracts were harvested after lysing in RIP lysis buffer (Solarbio, Beijing, China) and incubated with magnetic beads (Invitrogen) conjugated to antibodies of anti-Ago2 (Millipore) and anti-IgG (Millipore). RNA precipitates were extracted for qRT-PCR.

### RNA pull down assay

Cell lysate of Caki-1 or 786-O cells were obtained using lysis buffer (Invitrogen) and were then incubated with biotinylated RNAs, following by incubation with streptavidin-coupled magnetic beads (Invitrogen). After the binding RNAs were washed and eluted, qRT-PCR was conducted to analyze the expression of RNAs.

### TUNEL staining assay

The Cell Death Detection Kit (Roche) was chosen to detect the apoptosis level. After TUNEL staining, cells were respectively dyed using DAPI (Thermo Fisher Scientific). Relative fluorescence intensity was observed by the confocal laser scanning microscope (CLSM; Leica Microsystems, Berlin, Germany).

### Western blotting

Cells were lysed through RIPA buffer (Invitrogen). After centrifugation, the collected proteins were separated by SDS-PAGE (Bio-Rad, Hercules, CA, USA) and then transferred to polyvinylidene fluoride membranes (PVDF; Bio-Rad). The membranes were blocked with 5% nonfat milk and consecutively probed with primary antibodies including anti-γH2AX (ab2893, Abcam, Cambridge, USA), anti-cleaved PARP (ab32064, Abcam), anti-CHEK2 (ab109413, Abcam), anti-FOXM1 (ab180710, Abcam) and anti-GAPDH (ab245356, Abcam) as an internal control, followed by treated with secondary antibody. Finally, immunoreactivity was assessed via enhanced chemiluminescence (ECL; Bio-Rad).

### Luciferase reporter assay

LINC01094-WT, CHEK2-WT and their respective mutations (LINC01094-MUT, CHEK2-MUT) were synthesized by Genepharma (Shanghai, China) and inserted into the pmirGLO dual-luciferase plasmid (Promega, Madison, WI, USA). Then, LINC01094-WT/MUT vector and CHEK2-WT/MUT vector were co-transfected into HEK-293T cells with indicated transfection plasmids, respectively. After 48 h of transfection, Dual Luciferase Report Assay System (Promega) was applied to monitor luciferase activity.

### Statistical analysis

Experiments mentioned in this study were conducted for three times or more. All data were shown as mean ± standard deviation (SD). Statistics analysis was performed on GraphPad 5.0 software (GraphPad Software, La Jolla, CA, USA). Differences were compared using Student’s t-test or one-way ANOVA, with P < 0.05 used as significance threshold.

## Results

### Depletion of LINC01094 improves the sensitivity of ccRCC cells to radiation

**Fig. 1:**
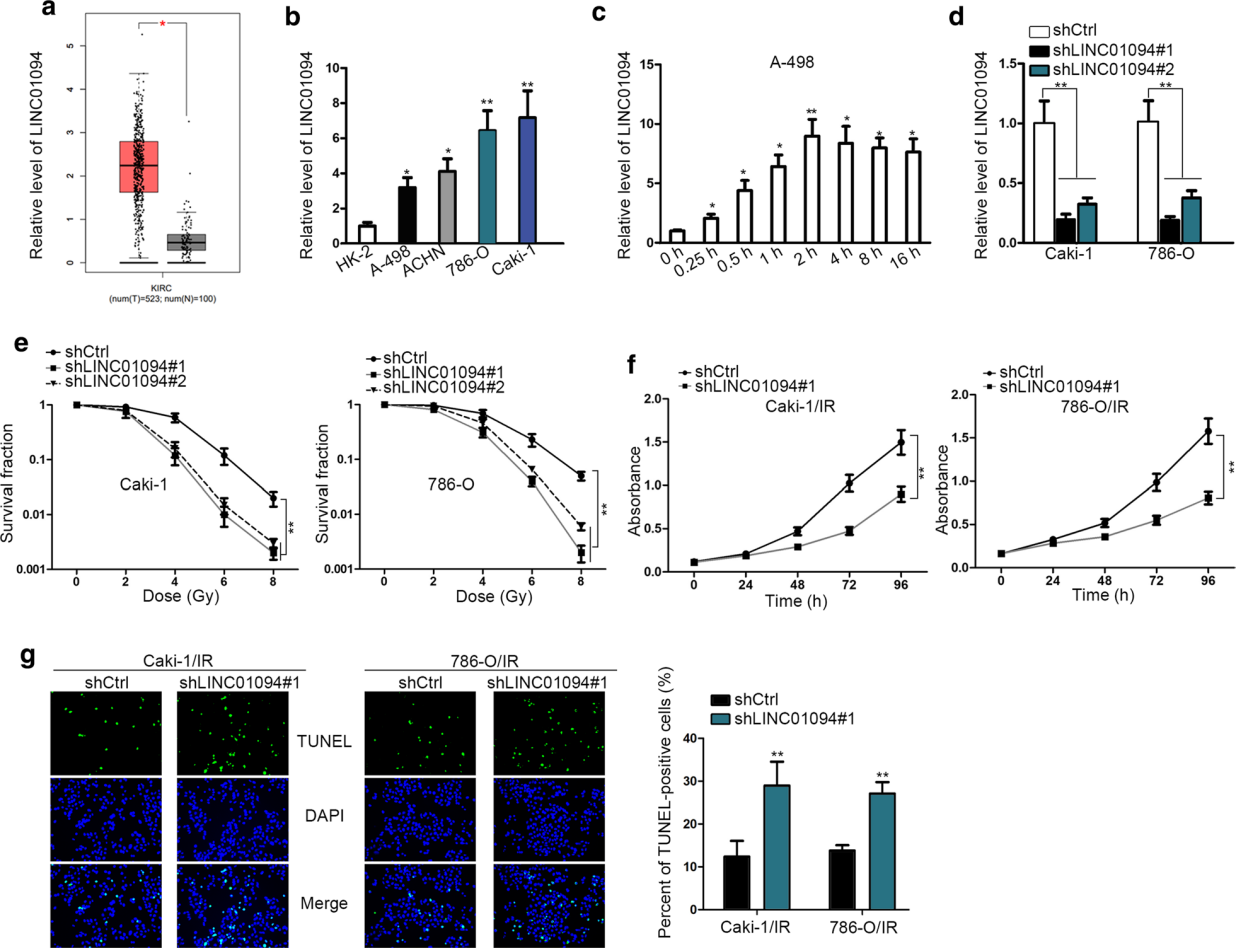
Upregulated LINC01094 accelerated tumorigenesis in ccRCC. **a** TCGA indicated the high expression of LINC01094 in KIRC tissues. **b** qRT-PCR analysis of relative expression of LINC01094 in ccRCC cell lines and normal HK-2 cells. **c** The expression level of LINC01094 in A-498 cells during irradiation exposure was examined via qRT-PCR. **d** The transfection efficiency of shRNAs against LINC01094 in Caski and 786-O cells was analyzed by qRT-PCR. **e** The survival fraction of above two ccRCC cells after transfections was determined by colony formation assay. **f** The impact of LINC01094 on the viability of ccRCC cells under exposure was estimated by MTT assay. **g** Cell apoptosis in irradiated Caski and 786-O cells with or without LINC01094 knockdown was evaluated through performing TUNEL assay. *P < 0.05, **P < 0.01

First of all, based on the TCGA data, we found that LINC01094 was obviously upregulated in KIRC tissues compared to the normal tissues (Fig. 1a). Also, it was demonstrated that LINC01094 was highly expressed in ccRCC cell lines (Fig. 1b). More importantly, LINC01094 was gradually upregulated in response to radiation exposure, and the final steady level was approximately 8 times of that under unexposed condition (Fig. 1c). On this basis, we suspected that LINC01094 might play a role in ccRCC radio-resistance development.

In order to verify above assertion, loss-of-function assays were performed subsequently. As a result, LINC01094 was apparently silenced in both Caki-1 and 786-O cells by transfection of shRNAs targeting LINC01094 (Fig. 1d). Depletion of LINC01094 consequently resulted in enhanced sensitivity of both Caki-1 and 786-O cells responding to radiation, evidenced by impaired survival fraction in LINC01094-inhibited ccRCC cells under radiation (Fig. 1e). Besides, considering Caki-1 and 786-O cells had worse survival fraction after being transfected with shLINC01094#1, we selected shLINC01094#1-transfected cells for following assays. Moreover, through being exposed to 4 Gy of radiation, the proliferative ability was notably decreased in ccRCC cells upon LINC01094 depletion (Fig. 1f). Meanwhile, knockdown of LINC01094 led to markedly intensified apoptosis in these two ccRCC cells under radiation exposure (Fig. 1g). Therefore, we concluded that LINC01094 upregulation leads to strengthened resistance of ccRCC cells responding to irradiation.

### LINC01094 confers radio-resistance in ccRCC through enhancing DNA-DSBs repair

**Fig. 2:**
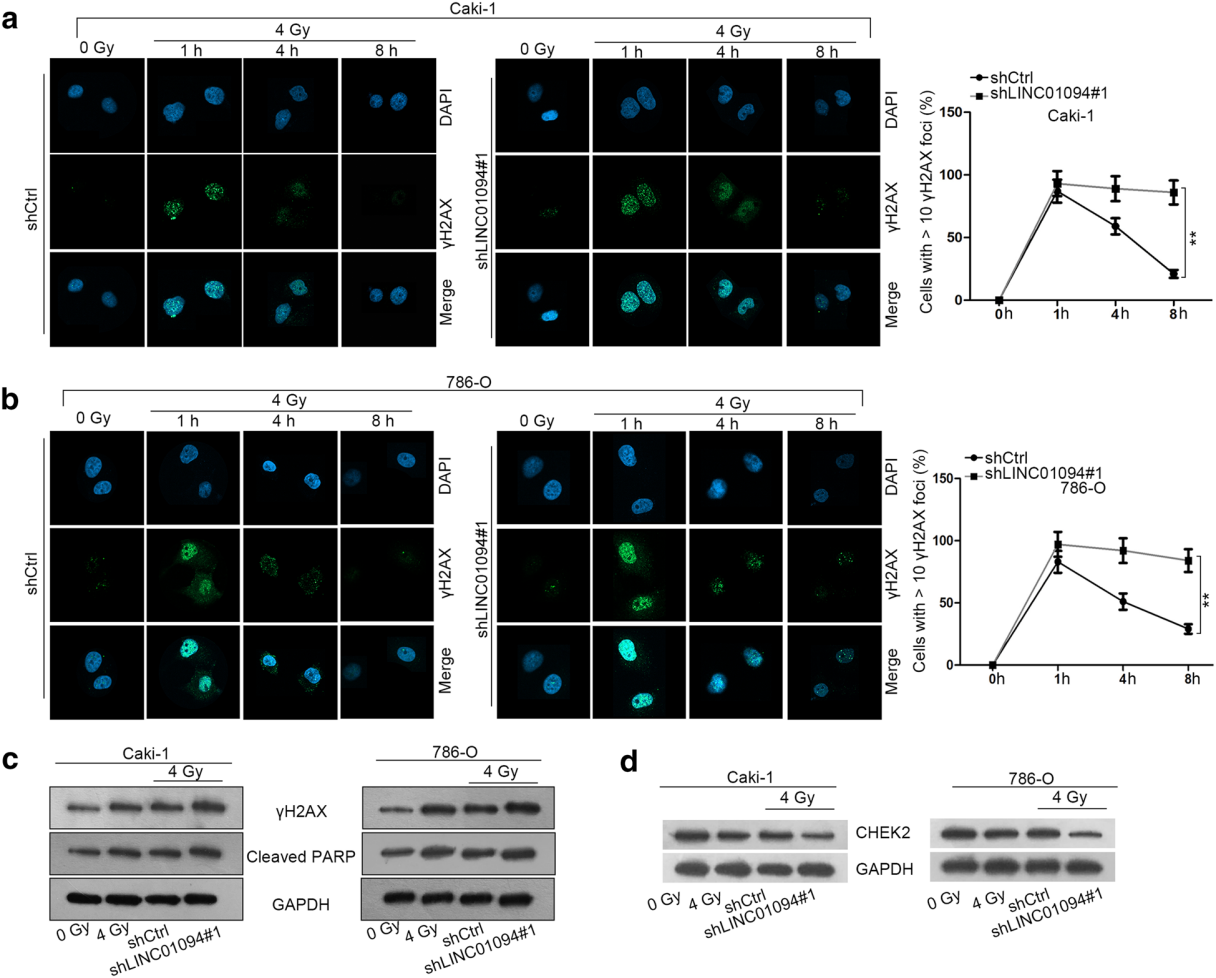
Inhibition of LINC01094 impaired the repair ability of ccRCC cells to DNA DSBs. **a**, **b** IF staining of γH2AX in Caski and 786-O cells responding to various conditions. **c**, **d** The level of several proteins regarding DNA DSBs repair in ccRCC cells was assessed by western blot. **P < 0.01

As is known, ionizing radiation (IR) confines tumor growth mainly depending on its induction of DNA damage and cell response to such damage is the key factor for tumor progression under radiotherapy [10]. Hence, we wondered whether LINC01094 influenced ccRCC radiosensitivity via affecting the repair of DNA DSBs. As expected, LINC01094 downregulation gave rise to delayed DNA DSBs repair but promoted DNA damage in ccRCC cells (Fig. 2a, b). Conformably, the protein level of γH2AX and cleaved PARP was much higher in cells with 4 Gy of irradiation compared to those with no exposure; more importantly, such enhancement was strengthened upon LINC01094 silence (Fig. 2c). In the meantime, the level of checkpoint kinase 2 (CHEK2) protein, a pivotal factor involving in DNA damage repair, was verified to be distinctly reduced under irradiated condition and was further downregulated by LINC01094 inhibition (Fig. 2d) Taken together, LINC01094 contributes to radiotolerance in ccRCC via improving DNA DSBs repair.

### LINC01094 locates mainly in the cytoplasm and sponges miR-577 in ccRCC

**Fig. 3:**
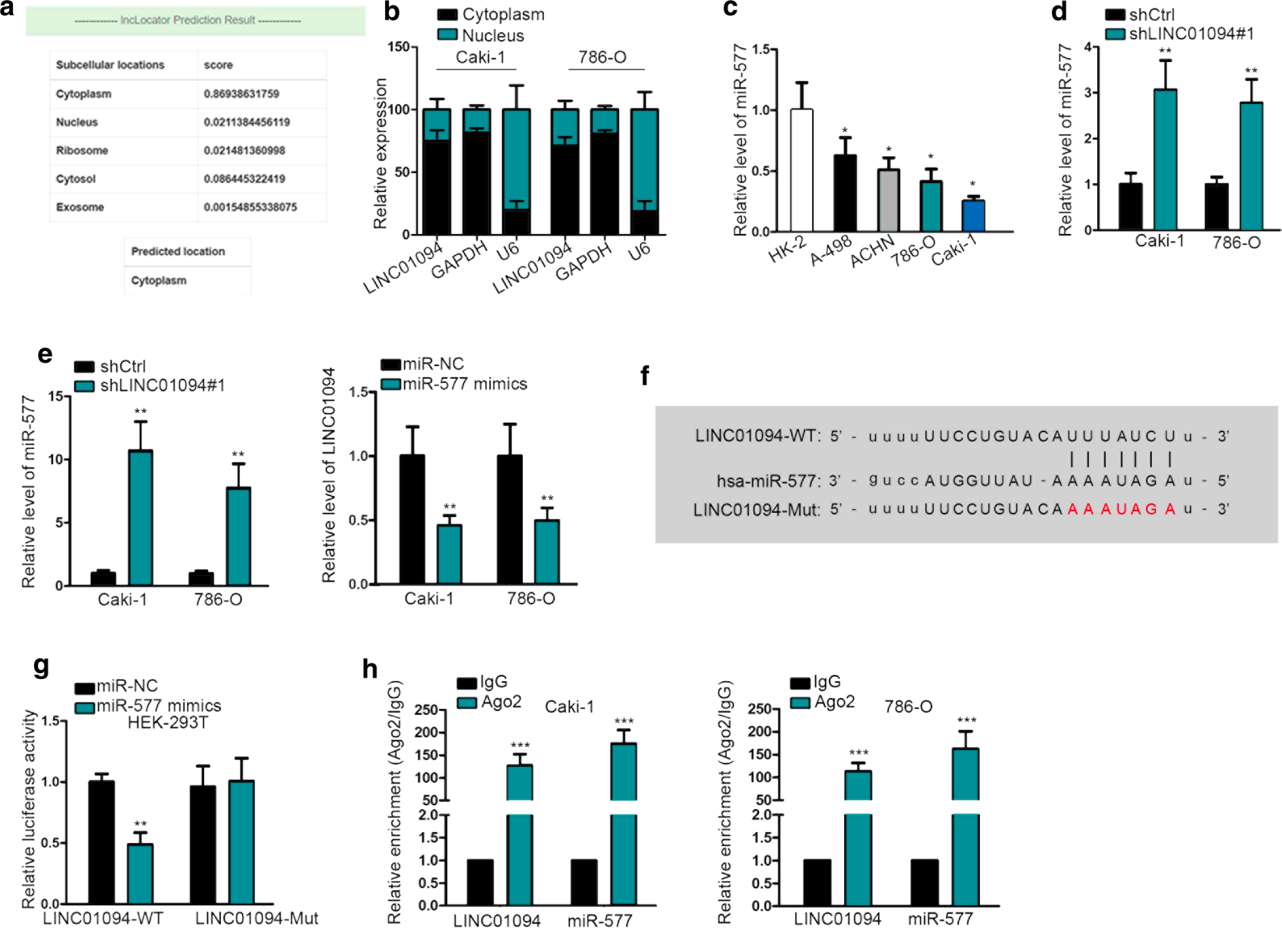
Cytoplasmic LINC01094 was an endogenous sponge of miR-577 in ccRCC. **a** lncLocator prediction of LINC01094 localization. **b** The specific distribution of LINC01094 in Caski and 786-O cells was determined via subcellular fractionation and qRT-PCR. **c** The expression of miR-577 in these ccRCC cell lines was tested by qRT-PCR. **d** qRT-PCR result of miR-577 level in Caski and 786-O cells with or without LINC01094 inhibition. **e** The expression of miR-577 or LINC01094 in Caski and 786-O cells with the transfection of miR-NC or miR-577 mimics was analyzed by qRT-PCR. **f**, **g** The binding sequences and results of luciferase reporter assay in HEK-293T cells. **h** RIP assay uncovered the co-enrichment of LINC01094 and miR-577 in Ago2-assembled RISC in two ccRCC cells. *P < 0.05, **P < 0.01, ***P < 0.001

Particularly, we probed into the underlying mechanism by which LINC01094 affected ccRCC radioresistance. Firstly, we explored the distribution of LINC01094 in ccRCC cells, since the function of lncRNAs depends on their subcellular localization to some extent. As a result, the online lncLocator suggested that most of LINC01094 located in the cytoplasm (Fig. 3a). Additionally, subcellular fractionation confirmed that LINC01094 was largely a cytoplasmic lncRNA in ccRCC (Fig. 3b). Given that lncRNAs in the cytoplasm largely function as a competing endogenous RNA (ceRNA) in cancers, we wondered whether LINC01094 was an endogenous sponge of certain miRNAs. After bioinformatics analysis, miR-577, miR-545-3p and miR-330-3p were suggested as potential miRNAs that interacted with LINC01094. According to the results of RNA pull down assay, only miR-577 was significantly pulled down by biotinylated LINC01094 (Additional file 1: Fig. S1A). Besides, miR-577 was downregulated in ccRCC cell lines in contrast to normal HK-2 cells (Fig. 3c). Moreover, we revealed that overexpression of miR-577 was stimulated by LINC01094 downregulation while the level of LINC01094 was mitigated under ectopic expression of miR-577 (Fig. 3d, e). Therefore, we speculated that LINC01094 might be a sponge of miR-577 in ccRCC.

To verify whether LINC01094 interacted with miR-577 in ccRCC cells, we constructed LINC01094-WT containing predicted binding sites of miR-577 and LINC01094-Mut containing mutated binding sites for luciferase reporter assay (Fig. 3f). As a consequence, the luciferase activity of LINC01094-WT was lessened under miR-577 upregulation, with that of LINC0194-Mut unchanged all the way (Fig. 3g). Furthermore, the RIP result certified that both LINC01094 and miR-577 were harvested by anti-Ago2 (Fig. 3h), revealing the interaction between them in RNA-induced silencing complex (RISC). Based on these data, we concluded that LINC01094 locates in the cytoplasm and interacts with miR-577 in ccRCC.

### LINC01094 increases FOXM1 protein in ccRCC by acting as a ceRNA of CHEK2 via absorbing miR-577

**Fig. 4:**
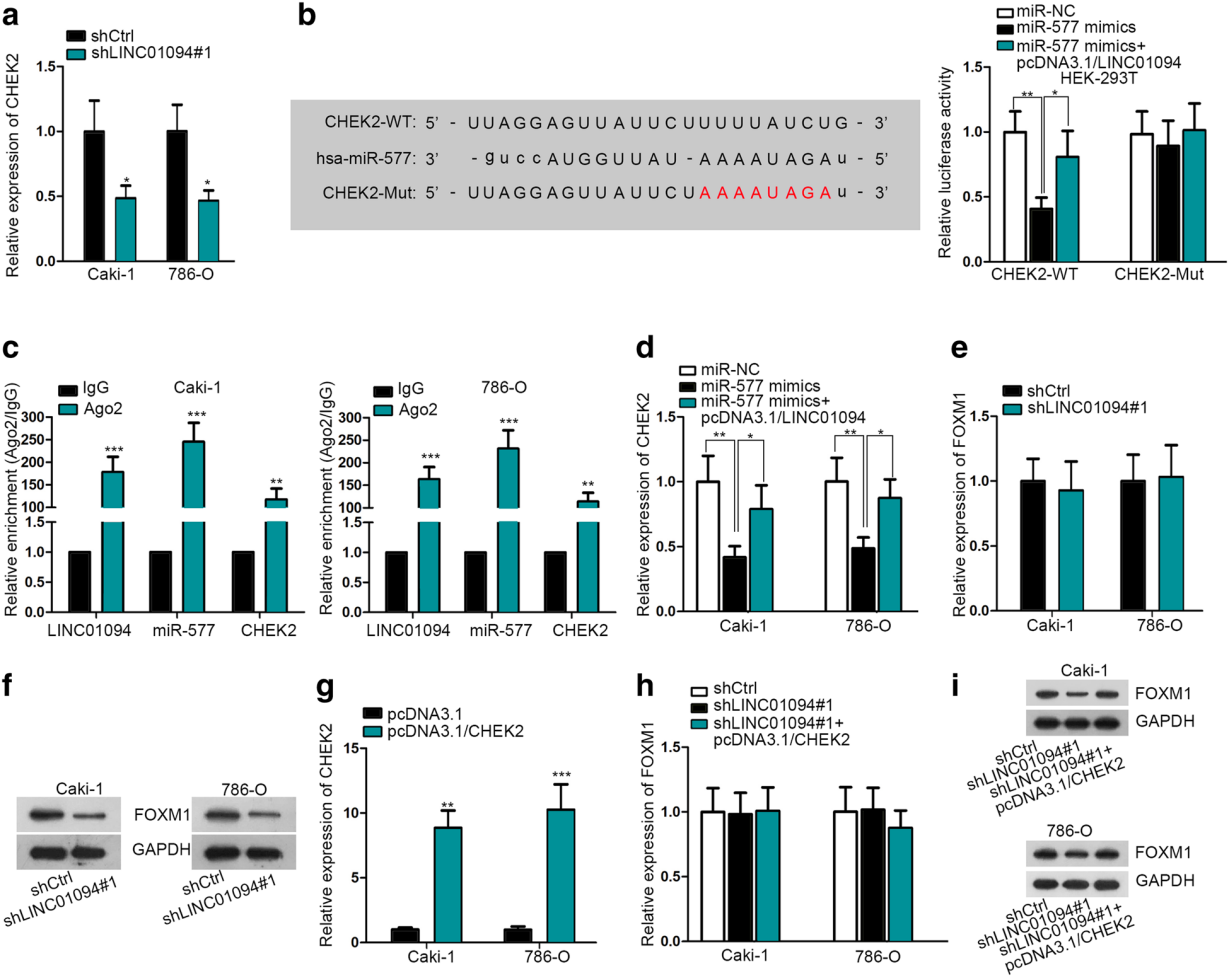
LINC01094 enhanced FOXM1 protein expression in ccRCC via miR-577/CHEK2 signaling. **a** qRT-PCR analysis of CHEK2 level in LINC01094-inhibited ccRCC cells. **b** Luciferase reporter assay confirmed the competitive binding of LINC01094 and CHEK2 mRNA with miR-577. **c** Co-existence of LINC01094, miR-577 and CHEK2 mRNA in RISC was validated through RIP assay. **d** The expression level of CHEK2 in indicated ccRCC cells was examined via qRT-PCR. **e**, **f** The expression of FOXM1 at mRNA and protein levels in Caski and 786-O cells with or without LINC01094 suppression was determined by qRT-PCR and western blot analyses. **g** Transfection efficiency of pcDNA3.1/CHEK2 and pcDNA3.1 was tested by qRT-PCR. **h**, **i** The mRNA and protein expression of FOXM1 in indicated ccRCC cells was detected via qRT-PCR and western blot. *P < 0.05, **P < 0.01, ***P < 0.001

Surprisingly, we discovered that CHEK2, a verified DNA damage-related gene, was predicted as one of the targets of miR-577 according to “ENCORI” database (http://starbase.sysu.edu.cn/). Importantly, the expression of CHEK2 was distinctly decreased by LINC01094 suppression (Fig. 4a), further implying that CHEK2 might be the downstream effector of LINC01094 in ccRCC. Moreover, we mutated predicted binding sequences of CHEK2 and conducted the following RNA pull down assay and luciferase reporter assay. The RNA pull down assay validated the interaction between miR-577 and CHEK2 at predicted sites (Additional file 1: Fig. S1B). And luciferase reporter assay disclosed that luciferase activity of CHEK2-WT was reduced by miR-577 mimics but was partially recovered by LINC01094 co-overexpression while CHEK2-MuT showed no response under the same conditions (Fig. 4b). Also, the coexistence of LINC01094, miR-577 and CHEK2 mRNA in RISC was testified in RIP assays (Fig. 4c). Consistently, the expression of CHEK2 was impeded by ectopic expression of miR-577, whereas such inhibition was attenuated under enforced LINC01094 expression (Fig. 4d). In other words, LINC01094 prompts CHEK2 expression in ccRCC via competitively binding with miR-577.

Forkhead box M1 (FOXM1) is an oncogenic transcription factor that is always upregulated in various cancers including ccRCC [19, 20]. Recently, FOXM1 has been reported to contribute to radioresistance in several carcinomas [21, 22]. Meanwhile, it has also been demonstrated that FOXM1 was stabilized by CHEK2 and therefore enhanced the expression of FOXM1-targeted DNA repair genes [23]. In this regard, we presumed that FOXM1 might be the downstream gene of LINC01094/miR-577/CHEK2 axis involved in ccRCC radio-sensitivity regulation. As anticipated, inhibition of LINC01094 had no impact on the mRNA level of FOXM1 but led to a sharp reduction on the protein level of FOXM1 in both Caki-1 and 786-O cells (Fig. 4e, f). Moreover, LINC01094 silence-confined FOXM1 protein level was recovered by CHEK2 upregulation, while the level of FOXM1 mRNA was scarcely influenced under the same condition (Fig. 4g–i). By and large, LINC01094 relies on CHEK2 to upregulate FOXM1 protein in ccRCC.

### FOXM1 mediates LINC01094-facilitated radioresistance in ccRCC

**Fig. 5:**
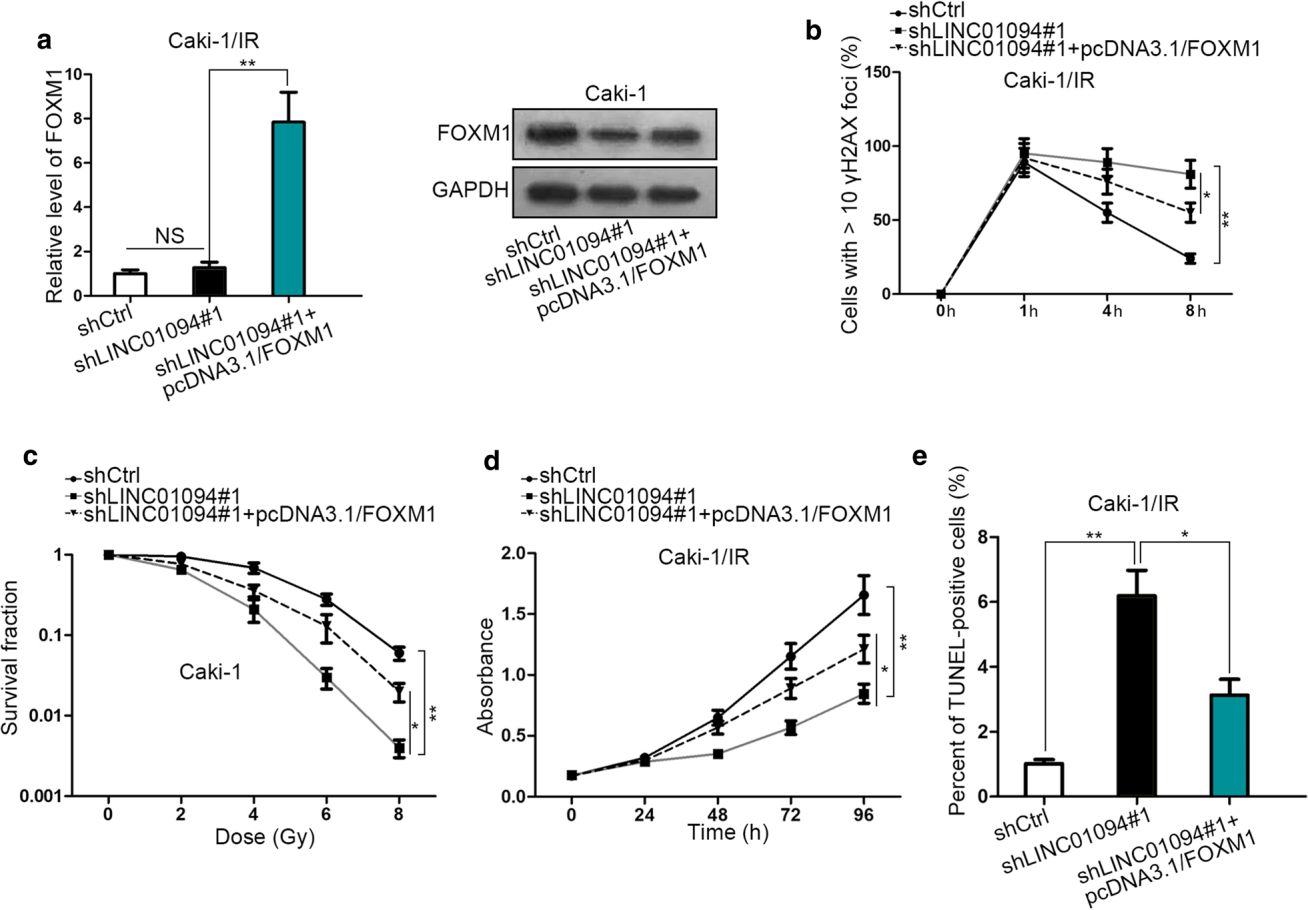
Enforced FOXM1 expression countervailed the enhancement of LINC01094 depletion on radiosensitivity of ccRCC cells. **a** Impact of pcDNA3.1/FOXM1 transfection on FOXM1 expression in LINC01094-silenced Caski cells was assessed by qRT-PCR and western blot. **b** IF staining of γH2AX foci in Caski cells responding to diverse transfections. **c**–**e** The survival fraction, viability and apoptosis of indicated Caski cells were respectively analyzed via performing colony formation assay, MTT assay and TUNEL assay. *P < 0.05, **P < 0.01

Subsequently, we attempted to make it clear that if FOXM1 was responsible for LINC01094-promoted DNA damage repair and consequent decreased radiosensitivity. As demonstrated in Fig. 5a, LINC01094 knockdown-restrained FOXM1 protein was reversed under the FOXM1 overexpression. In addition, upregulation of FOXM1 recovered the DNA DSBs repair controlled by LINC01094 suppression in Caki-1 cells under irradiation (Fig. 5b). Moreover, lessened cell survival fraction, viability as well as accelerated cell apoptosis of 4 Gy radiation-exposed cells under LINC01094 silence were all normalized by FOXM1 upregulation (Fig. 5c–e). All these assays proved that heightened sensitivity of Caki-1 cells to radiation exposure under LINC01094 depletion was restored upon enforced FOXM1 expression. Hence, we drew a conclusion that LINC01094 contributes to DNA DSBs repair to improve ccRCC radioresistance through FOXM1-responsible manner.

## Discussion

Nowadays, radiotherapy has been commonly employed to treat more than half of human cancer types [24, 25], including renal cell carcinoma [26]. Nevertheless, some radioresistance-obtained cells, namely cancer stem-like cells, has become the major obstacle to control the efficacy of radiotherapy [27]. To overcome radioresistance, great efforts have been made over decades. Recently, lncRNAs have been recognized as novel regulators in radioresistance development [17, 28, 29]. Here, we studied on a novel lncRNA LINC01094, which was suggested by TCGA to be highly-expressed in KIRC tissues Expression of LINC01094 was ever-increasing till reaching a stable level (8 times of the inital level) in ccRCC cells during radiation exposure. On this basis, we assumed that LINC01094 might be associated with radioresistance development in ccRCC. As expected, knockdown of LINC01094 led to increased sensitivity of ccRCC cells to radiation treatment, indicating the promotion effects of LINC01094 on ccRCC radioresistance. A recent study uncovered the implication of ncRNAs including lncRNAs in DNA repair in radiation-exposed tumor development [13]. Ionizing radiation can induce a wide range of DNA lesions and clustered DNA lesions is the hallmark of IR. DNA double strand breaks (DSBs) play a key role during the process [30, 31]. Presently, we revealed that LINC01094 affected the radioresistance in ccRCC via promoting ability of ccRCC cells to repair DNA DSBs.

In recent years, lncRNAs in the cytoplasm have been emerged as competing endogenous RNAs in regulating cancer development via sponging certain miRNA to release such miRNAs-targeted mRNAs from degradation at post-transcriptional level [32, 33]. Herein, it was proved that LINC01094 was mainly a cytoplasmic lncRNA in ccRCC cells. More importantly, we demonstrated that miR-577, which was predominantly reported as an anti-tumor miRNA [34, 35], was directly sponged by LINC01094 in ccRCC. Further, CHEK2 (also called Chk2), a well-known factor that participated in DNA repair and radioresitance development [36–38], was recognized as the downstream target of miR-577 and had a competition with LINC01094 to interact with miR-577 in ccRCC cells. Besides, we validated that LINC01094 enhanced FOXM1 protein level in ccRCC at CHEK2-dependent manner, which was in consistent with a previous study that CHEK2 stabilized FOXM1 protein in stimulation of DNA repair-related genes [23]. Last but not least, rescue assays conducted in LINC01094-depleted ccRCC cells further certified that FOXM1 was the responsible effector underlying LINC01094-strengthened radioresistance in ccRCC.

## Conclusion

All in all, our work illustrated that LINC01094 contributes to radioresistance development in ccRCC through modulating CHEK2-stabilized FOXM1 by acting as a ceRNA to up-regulate CHEK2, which might open up a new way to overcome the radioresistance in ccRCC.

## Supplementary information

**Additional file 1: Figure S1.** (A-B) RNA pull down assay detected relative expression of miRNAs pulled down by different biotinylated RNAs. **P < 0.01.

## Data Availability

Not applicable.

## Abbreviations

RCC: Renal cell carcinoma
lncRNAs: Long non-coding RNAs
ccRCC: Clear cell renal cell carcinoma
DSBs: DNA double-strand breaks
IR: Ionizing radiation
LINC01094: Long intergenic non-protein coding RNA 1094
KIRC: Kidney renal clear cell carcinoma
ATCC: American Type Culture Collection
FBS: Fetal bovine serum
qRT-PCR: Quantitative real-time PCR
IF: Immunofluorescence
DAPI: 4′,6-diamidino-2-phenylindole
RIP: RNA immunoprecipitation
CLSM: The confocal laser scanning microscope
PVDF: Polyvinylidene fluoride membranes
ECL: Enhanced chemiluminescence
SD: Standard deviation
CHEK2: Checkpoint kinase 2
ceRNA: Competing endogenous RNA
RISC: RNA-induced silencing complex
FOXM1: Forkhead box M1
