# Supplementary figures and images for: LINC01094 triggers radio-resistance in clear cell renal cell carcinoma via miR-577/CHEK2/FOXM1 axis
Source: Cancer Cell Int. 2020 Jun 24;20:274. doi: 10.1186/s12935-020-01306-8 (PMC7315499; doi:10.1186/s12935-020-01306-8)

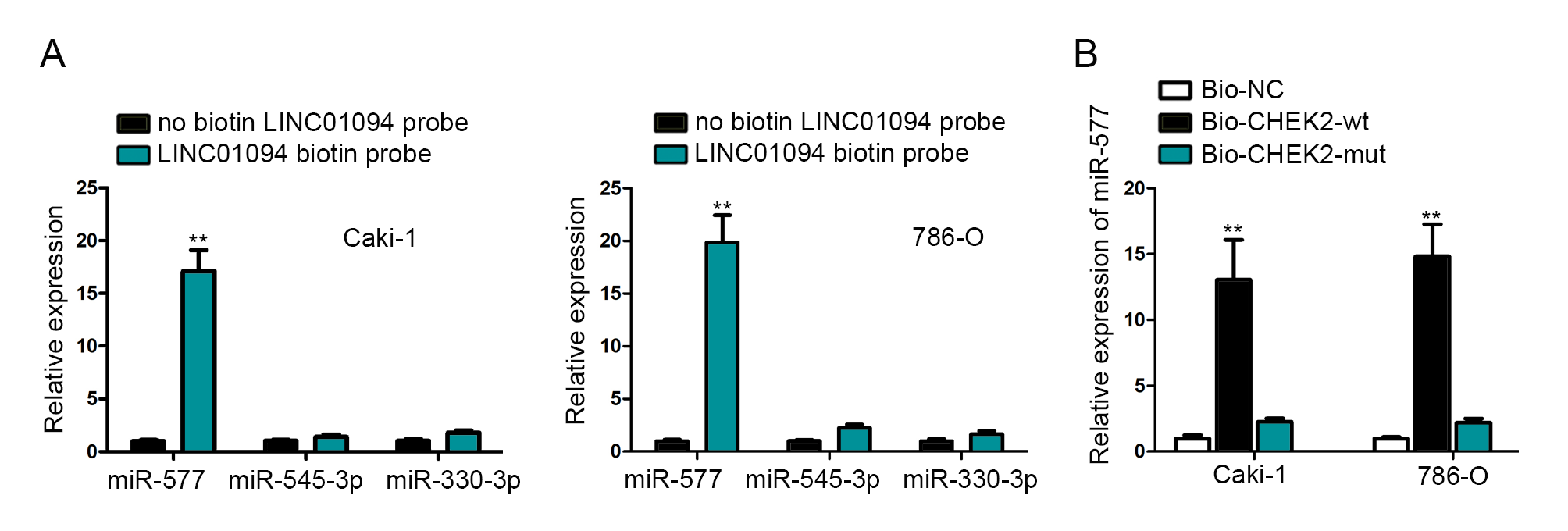

Supplement: Supplementary file 1 — Additional file 1: Figure S1. (A-B) RNA pull down assay detected relative expression of miRNAs pulled down by different biotinylated RNAs. **P < 0.01. [file 12935_2020_1306_MOESM1_ESM.tif]
